# Association between short-term exposure to ambient air pollution and heart failure: An updated systematic review and meta-analysis of more than 7 million participants

**DOI:** 10.3389/fpubh.2022.948765

**Published:** 2023-01-23

**Authors:** Yu-shan Yang, Ying-hao Pei, Yuan-yuan Gu, Jun-feng Zhu, Peng Yu, Xiao-hu Chen

**Affiliations:** ^1^First Clinical Medical College, Nanjing University of Chinese Medicine, Nanjing, China; ^2^Department of Cardiology, Jiangsu Province Hospital of Chinese Medicine, Affiliated Hospital of Nanjing University of Chinese Medicine, Nanjing, Jiangsu, China; ^3^Department of Intensive Care Unit, Jiangsu Province Hospital of Chinese Medicine, Affiliated Hospital of Nanjing University of Chinese Medicine, Nanjing, China

**Keywords:** heart failure, air pollution, particulate matter, gas pollutant, meta-analysis

## Abstract

**Introduction:**

Exposure to air pollution has been linked to the mortality of heart failure. In this study, we sought to update the existing systematic review and meta-analysis, published in 2013, to further assess the association between air pollution and acute decompensated heart failure, including hospitalization and heart failure mortality.

**Methods:**

PubMed, Web of Science, EMBASE, and OVID databases were systematically searched till April 2022. We enrolled the studies regarding air pollution exposure and heart failure and extracted the original data to combine and obtain an overall risk estimate for each pollutant.

**Results:**

We analyzed 51 studies and 7,555,442 patients. Our results indicated that heart failure hospitalization or death was associated with increases in carbon monoxide (3.46% per 1 part per million; 95% CI 1.0233–1.046, *P* < 0.001), sulfur dioxide (2.20% per 10 parts per billion; 95% CI 1.0106–1.0335, *P* < 0.001), nitrogen dioxide (2.07% per 10 parts per billion; 95% CI 1.0106–1.0335, *P* < 0.001), and ozone (0.95% per 10 parts per billion; 95% CI 1.0024–1.0166, *P* < 0.001) concentrations. Increases in particulate matter concentration were related to heart failure hospitalization or death (PM_2.5_ 1.29% per 10 μg/m^3^, 95% CI 1.0093–1.0165, *P* < 0.001; PM_10_ 1.30% per 10 μg/m^3^, 95% CI 1.0102–1.0157, *P* < 0.001).

**Conclusion:**

The increase in the concentration of all pollutants, including gases (carbon monoxide, sulfur dioxide, nitrogen dioxide, ozone) and particulate matter [(PM_2.5_), (PM_10_)], is positively correlated with hospitalization rates and mortality of heart failure.

**Systematic review registration:**

https://www.crd.york.ac.uk/PROSPERO/, identifier: CRD42021256241.

## Introduction

Heart failure (HF) is a group of clinical syndromes characterized by dyspnea or fatigue caused by ventricular filling or ejection disorders or both (1), which is the ultimate destination of all cardiovascular diseases and is the leading cause of global morbidity and mortality (2). The incidence of HF is rising steadily worldwide, but the prognosis is still poor (3). More than 64 million people are suffering from HF in the world, with an estimated prevalence of 1–2% among adults in developed countries (4).

Air pollution is a worldwide problem affecting human health (5). It has been recognized as an independently detrimental factor to respiratory and cardiovascular diseases (6). According to the report of the World Health Organization, almost 7 million people die due to air pollution every year, indicating air pollution to be the world's largest environmental health risk (7). Particulate matters (PM) and gases are the main components of air pollution that have been reported to induce stroke (8), myocardial infarction (9), HF, atrial fibrillation, and sudden cardiac death (10) in multiple populations. Air pollution-induced cardiovascular events have received more attention than other related toxicity, such as pulmonary diseases (11). Epidemiological studies suggest an association between short-term fluctuations in ambient air pollution and the risk of hospitalization for acute cardiovascular events, including acute decompensated HF (12). In 2013, Shah et al. (13) reviewed and meta-analyzed the studies on air pollution and HF published from 1948 to 2012. The results showed that the increase in the concentration of all pollutants, except ozone, is positively related to the incidence and mortality of HF patients. However, all studies, but one in this meta-analysis, were conducted in developed countries. Moreover, over 22 studies, including 4 million participants and 14 studies in developing countries, focusing on the relationship between air pollution and HF have been published after Shah's study (13).

Thus, we updated the systematic review and meta-analysis to reassess the relationship between air pollutants and HF outcomes (hospitalization and death). This systematic review and meta-analysis were performed according to the guidelines of the Preferred Reporting Items for Systematic Review and Meta-analyses (PRISMA) criteria (Supplementary Table S1). This meta-analysis has been registered with PROSPERO (ID: CRD42021256241).

## Methods

### Search strategy

Literature was searched in four databases, including PubMed, Web of Science, EMBASE, and OVID databases, from their inception until 30 April 2022. The following keywords were included for the search: “heart failure,” “air pollution,” “particulate matter,” “ozone,” “carbon monoxide,” “sulfur dioxide,” “nitrogen dioxide,” and “carbon dioxide.”

First, we performed a preliminary screening of the titles and abstracts. Then, we further evaluated the full texts of potentially eligible studies. We manually searched the reference lists of all the included studies. Literature selection and study quality assessment were completed by two independent authors (Y.Y. and Y.G.), and conflicts between the two authors were resolved after a discussion with an arbitrator (Y.P.).

### Inclusion and exclusion criteria

Articles that met the following criteria were included: (1) original human epidemiological studies reported associations between air pollution exposure and HF hospitalization rate or mortality up to and including lag (day) 7, confidence interval (95%CI); (2) case-crossover, time series (both assessed by generalized linear regression models); (3) focused on exposure of outdoor (ambient) air pollution, but not of indoor air pollution; and (4) published in English. Studies were excluded if they were (1) animal or experimental studies; (2) case reports, comments, or reviews; or (3) studies that reported an unclear increment of air pollutant concentrations.

### Data extraction

Data were extracted from all the selected studies, including (1) study characteristics (first author, published year, study location, and period); (2) study populations (sample size and range of age); (3) outcomes [hospitalization rates and mortality, lag (days)]; (4) air pollution measurement method and increment of air pollution used in effect evaluation, including per interquartile range (IQR), standard deviation (SD), or per 10 μg/m^3^; and (5) effect estimates of the association between air pollution and HF (OR, RR, with 95% CI). The effect estimates of the single-pollutant model were extracted.

### Quality assessment

The Newcastle–Ottawa Quality Assessment Scale (NOS) checklist was applied to assess the quality of the studies enrolled in this study. Two authors (Y.Y. and Y.G.) worked independently, and inconsistencies in the quality assessment were resolved through discussion with an arbitrator (Y.P.). The score ranges from 0 to 9 points. A higher score indicates a higher study quality. A study with a score of ≥7 was regarded as high quality; otherwise, the study was considered low quality (14).

### Data synthesis

We synthesized the data extracted from the included studies by the following formula. The relative risk (RR) is combined into a standardized pollutant concentration increment before the meta-analysis as follows: 10 μg/m3 for PM_2.5_ and PM_10_; 10 parts per billion for nitrogen dioxide (NO_2_), sulfur dioxide (SO_2_), and ozone (O_3_); and 1 part per million for carbon monoxide (CO).


$$\begin{array}{l} \text{RR }\left(\text{standardized}\right)=\;\text{Increment (10)}\left(\text{original}\right)/\text{Increment }\left(\text{original}\right) \end{array}$$

Many studies provide multiple estimates of a single lag, such as lag 0 or lag 1, which are merged separately. The shortest lag is used to evaluate the overall risk estimate. A few studies provided only cumulative lag (e.g., lag 0–1 or lag 0–2), which is not suitable for merging in a single lag analysis but is used to determine the overall risk estimates.

The original study data were further stratified by study design (case-crossover vs. time series), age (all ages vs. 60 years), and outcome (hospitalization vs. mortality). Assuming the prevalence of air pollution exposure is 100%, we used our overall risk estimate and formula to calculate the population-attributable risk for each pollutant as follows:


$$\begin{array}{l} \text{Population}-\text{attributable risks}=\frac{\left(RR-\;1\right)}{RR} \end{array}$$

### Statistical analysis

Stata version 15.0 (StataCorp., College Station, TX, USA) software was used to calculate the impact of each pollutant on the HF hospitalization rate or mortality and the 95% CI. The significance of the pooled OR and RR was determined by the *Z* test (15), and a *p*-value of < 0.05 was considered statistically significant.

The heterogeneity test was performed by the standard *I*^2^ test. If *I*^2^ ≥ 50% (*P* ≤ 0.10), it was considered that there was heterogeneity among the studies, and then, the Dersimonian–Laird random-effects model was used to combine the values. If *I*^2^ < 50% (*P* > 0.10), the research was regarded as homogeneous, and then, the Mantel–Haenszel fixed-effects model was performed to merge values. We expected that the heterogeneity between studies was due to different study designs, analysis methods, different lagging exposures, and geographic and population differences.

The sensitivity analysis was performed by eliminating individual studies one by one to evaluate the stability of the results. The funnel plot and Egger linear regression were used to test for publication bias, and if there was publication bias, the cut-and-compensation method was used to correct the bias.

## Results

### Search process and study characteristics

**Figure 1:**
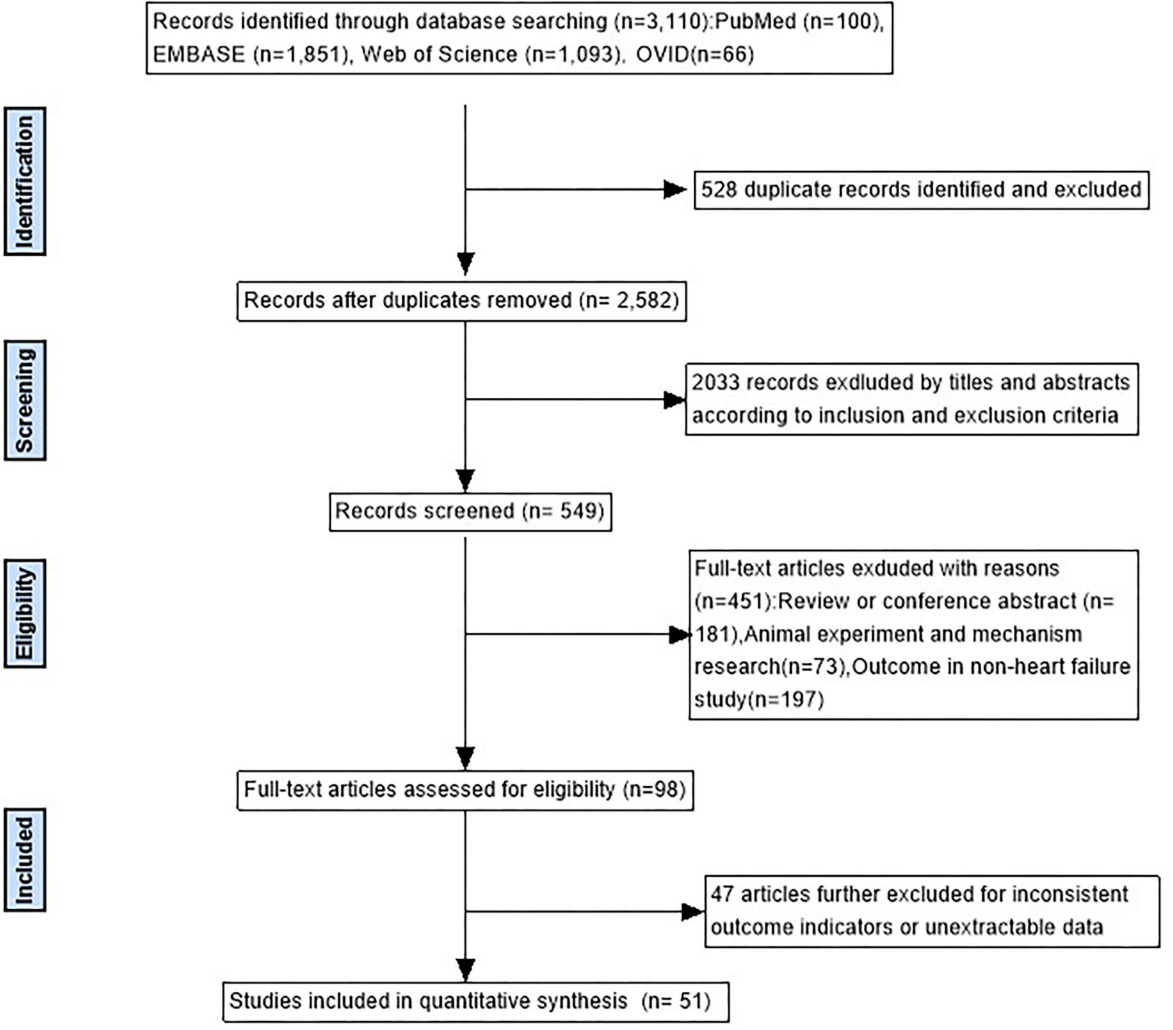
A flowchart of literature screening.

As can be seen in Figure 1, there were 3,110 studies enrolled in the initial search. After removing duplicates and screening titles, 549 studies underwent in-depth review, and 51 studies matched the inclusion criteria. In all, 30 studies used a time series design (16–45), 20 studies used a case-crossover design (46–65), and 1 used both study designs (66). Of the 51 studies, 19 were from developing countries and 32 from developed countries. Specifically, 21 are in the Americas (17 in the United States, 2 in Canada, and 2 in Brazil), 20 in Asia (16 in China, 1 in Thailand, 1 in South Korea, and 2 in Japan), 7 in Europe (4 in Italy, 2 in the United Kingdom, and 1 in the Netherlands), and 3 in Oceania (2 in Australia and 1 in New Zealand). The general characteristics of the studies included in the meta-analysis and details of the quality assessment are displayed in Table 1 and Supplementary Table S2, respectively.

**Table 1:** Contextual details of studies included in the meta-analysis.

**References**	**Location**	**Published**	**Period**	**Study design**	**Data source**	**Population**	**Number of events**	**Outcome**
Schwartz et al. (17)	USA	1995	1986–1989	Time-series	Medicare data	≥65 years	38,862	HA
Poloniecki et al. (18)	UK	1997	1987–1994	Time-series	Hospital episode records	≥65 years	62,853	HA
Morris et al. (19)	USA	1998	1986–1989	Time-series	Medicare data	≥65 years	49,640	HA
Wong et al. (20)	Hong Kong	1999	1994–1995	Time-series	Hospital data admission registry	All	NR	HA
Wong et al. (21)	Hong Kong	1999	1995–1997	Time-series	Hospital authority data	≥65 years	NR	HA
Hoek et al. (22)	Netherlands	2001	1986–1994	Time-series	Death certificates	All	45,333	Mortality
Kwon et al. (66)	South Korea	2001	1994–1998	Case-crossover and time-series	Mortality records	≥65 years	1,807	Mortality
Ye et al. (23)	Japan	2001	1980–1995	Time-series	Ministry of Health	≥65 years	4,469	HA
McGowan et al. (24)	New Zealand	2002	1988–1998	Time-series	Hospital data admission registry	All	5,146	HA
Goldberg et al. (25)	Canada	2003	1984–1993	Time-series	Billing and prescription data	≥65 years	16,794	Mortality
Koken et al. (26)	USA	2003	1993–1997	Time-series	Agency for Healthcare Research and Quality	All	1,860	HA
Bateson et al. (46)	USA	2004	1988–1991	Case-crossover	Medicare and medicaid data	All	26,923	Mortality
Metzger et al. (27)	USA	2004	1993–2000	Time-series	Billing data	All	20,073	HA
Wellenius et al. (47)	USA	2005	1987–1999	Case-crossover	Medicare and medicaid data	≥65 years	55,019	HA
Martins et al. (28)	Brazil	2006	1996–2001	Time-series	Department of Data Analysis of the Unified Health System	≥65 years	24,476	HA
Dominici et al. (29)	USA	2006	1999–2002	Time-series	Medicare data	≥65 years	986,392	HA
Wellenius et al. (48)	USA	2006	1986–1999	Case-crossover	Medicare and Medicaid data	All	292,918	HA
Barnett et al. (49)	Australia and New Zealand	2006	1998–2001	Case-crossover	Government health departments (Australia) and Ministry of Health (NZ)	≥65 years	NR	HA
Lee et al. (30)	Taiwan	2008	1996–2004	Time-series	National Health Institute registry	All	13,475	HA
Peel et al. (50)	USA	2007	1993–2000	Case-crossover	Billing records	>64 years	20,073	HA
Forastiere et al. (51)	Italy	2008	1997–2004	Case-crossover	Regional registries of cause of death	All	9,569	Mortality
Yang et al. (52)	Taiwan	2008	1996–2004	Case-crossover	National Health Institute registry	All	24,240	HA
Bell et al. (31)	USA	2009	1999–2005	Time-series	Medicare data	All	1,142,928	HA
Haley et al. (53)	USA	2009	2001–2005	Case-crossover	NYSDOH registry	All	170,502	HA
Stieb et al. (32)	Canada	2009	1999–2000	Time-series	Emergency department registry	All	32,313	HA
Ueda et al. (33)	Japan	2009	2002–2004	Time-series	Ministry of Health	≥65 years	17,548	Mortality
Zanobetti et al. (34)	USA	2009	2000–2003	Time-series	Medicare data	All	238,587	HA
Colais et al. (54)	Italy	2012	2001–2005	Case-crossover	Hospital discharge registry	≥65 years	55,339	HA
Belleudi et al. (55)	Italy	2010	2001–2005	Case-crossover	Hospital discharge registry	≥65 years	17,561	HA
Hsieh et al. (56)	China	2013	2006–2010	case-crossover	The National Health Insurance	All	20,776	HA
Yang et al. (35)	China	2014	2008–2012	Time-series	Guangzhou Emergency Center	All	3,375	HA
Milojevic et al. (57)	UK	2014	2003–2009	Case-crossover	the Myocardial Ischaemia National Audit Project (MINAP) database	All	37,033	Mortality
Chen et al. (58)	China	2015	2006–2010	Case-crossover	The National Health Insurance	All	64,002	HA
Weber et al. (59)	USA	2016	2004–2006	Case-crossover	The New York State Planning and Research Cooperative System (SPARCS)	≥35	342,411	HA
Vaduganathan et al. (60)	Italy	2016	2004–2007	Case-crossover	The Hospital Discharge Database	All	6,000	HA
Dabass et al. (61)	USA	2016	1999–2011	Case-crossover	The Pennsylvania Department of Health Vital Statistics Division	All	4,358	Mortality
Xu et al. (36)	China	2017	2013	Time-series	Emergency department registry	All	56,221	HA
Liu et al. (62)	China	2018	2014–2015	Case-crossover	the National Hospital Performance Evaluation Project of the National Healthcare Data Center of China	≥18	105,501	HA
Hsu et al. (37)	USA	2017	1991–2006	Time-series	The NYS Department of Health's Statewide Planning and Research Cooperative System	All	999,264	HA
Li et al. (38)	China	2018	2010–2012	Time-series	Beijing Medical Claim Data for Employees	≥18	15,256	HA
Huynh et al. (39)	Australian	2018	2009–2012	Time-series	The Clinical Informatics and Business Intelligence Unit of the Department of Health and Human Services of Tasmania.	All	1,246	HA
Li et al. (63)	China	2018	2013–2017	Case-crossover	The Beijing Municipal Commission of Health and Family Planning Information Center	>18	58,393	HA
Zhang et al. (64)	USA	2018	2014–2016	Case-crossover	The inpatient SPARCS database	All	1,917,823	HA
Pothirat et al. (40)	Thailand	2019	2016–2017	Time-series	Hospital registry records	All	859	HA
Amsalu et al. (16)	China	2019	2013–2017	Time-series	the Beijing Public Health Information Center	≥18	58,432	HA
Tian et al. (41)	China	2019	2014–2017	Time-series	The Urban Employee Basic Medical Insurance (UEBMI)	≥18	4,383	HA
Wu et al. (42)	China	2019	2014–2017	time-series	China Center for Disease Control and Prevention	All	1,782	Mortality
Feng et al. (43)	China	2019	2013	time-series	The Beijing Medical Research Data	All	56,212	HA
Qiu et al. (65)	USA	2020	2000–2012	Case-crossover	Center for Medicare and Medicaid Services	>64	204,774	HA
Gu et al. (44)	China	2020	2013–2017	time-series	Hospital Quality Monitoring System of China	All	87,052	HA
Pamplona et al. (45)	Brazil	2020	2000–2013.	time-series	The Unified Health System database (DA TASUS)	>60	135,589	HA

HA, hospital admissions; NR, not report.

### Exposure to air pollution and the rate of HF hospitalization or mortality

**Figure 2:**
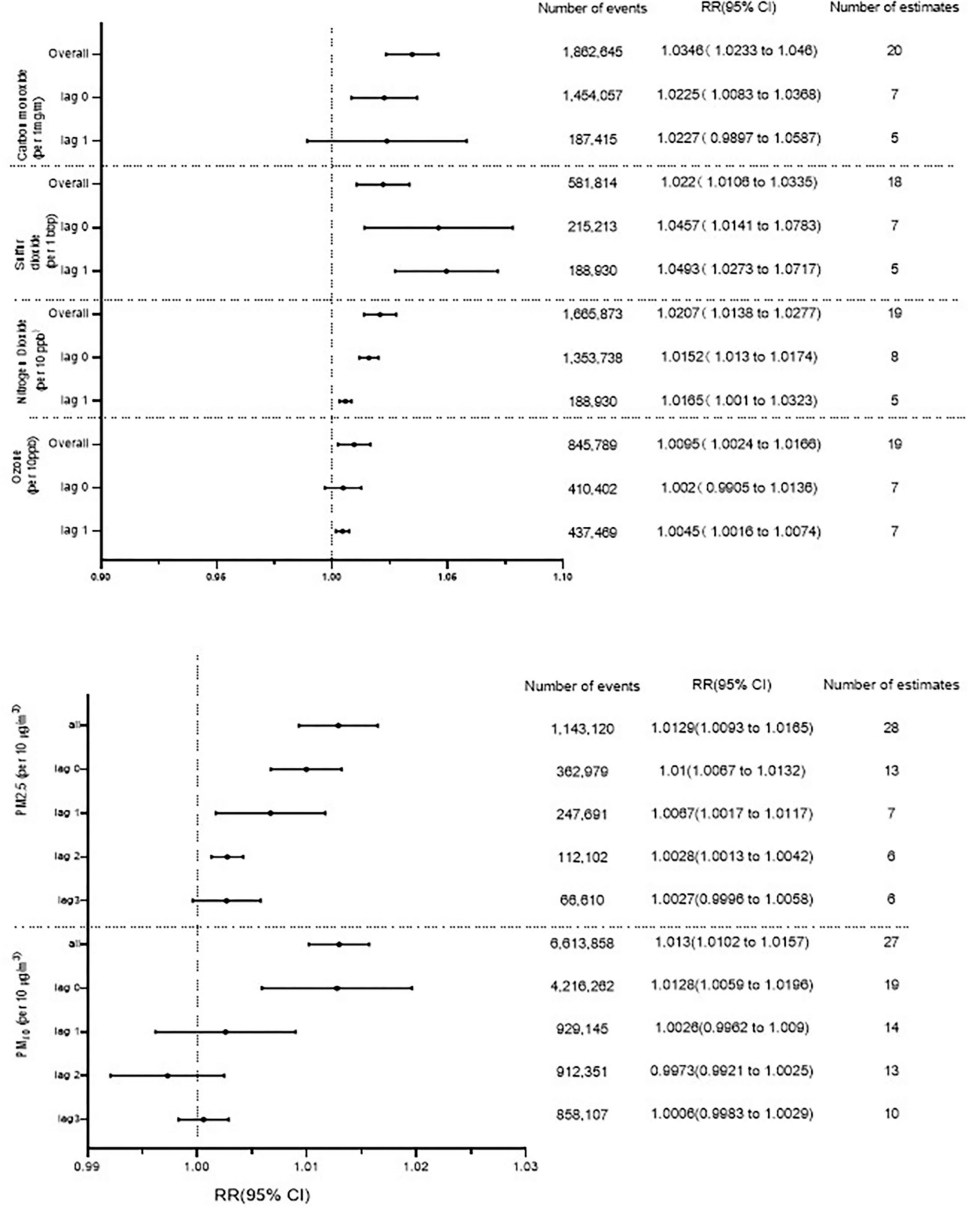
Association between gaseous and particulate air pollutants and heart failure hospitalization or heart failure mortality. ppm, parts per million; ppb, parts per billion.

**Figure 3:**
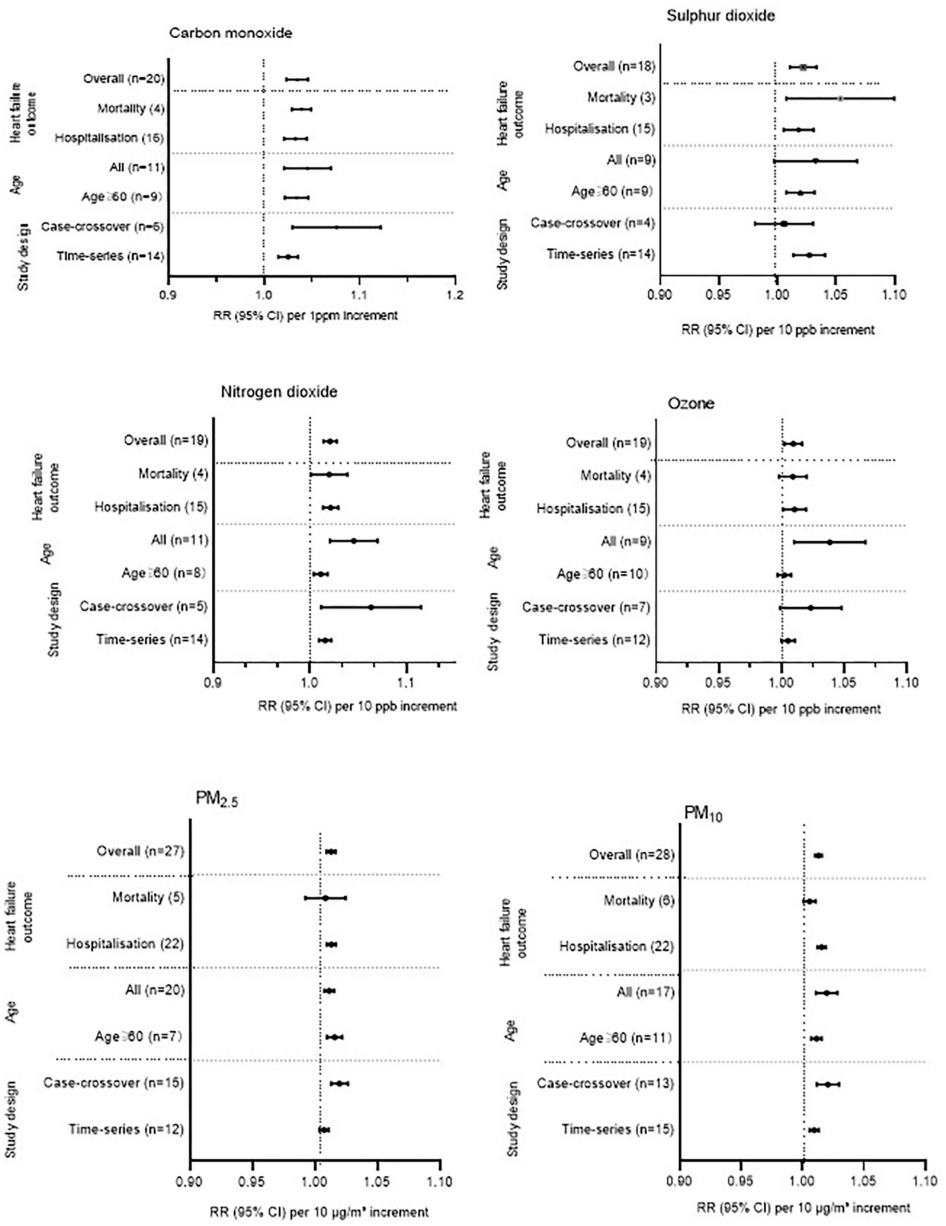
Additional analysis across all gaseous and particulate air pollutants. Some studies provided separate estimates for all age groups and for people older than 65 years. This study, therefore, appears two times in the additional analysis when stratified by age. For the overall analysis, we have used the estimates provided for all age groups. Some studies provided separate estimates stratified by study design and, therefore, appear twice in the additional analysis. For the overall analysis, we used the estimates provided for the time-series study design. ppm, parts per million; ppb, parts per billion.

As can be seen in Figure 2, HF hospitalization or mortality was positively correlated with all air pollution, which was consistent with the results of Shah's (13) meta-analysis, except O_3_. HF hospitalization or mortality was increased by 3.46% (95%CI 1.0233–1.046, *P* < 0.001) per increase of 1 part per million of CO. Each 10 ppb increase in SO_2_, O_3_, and NO_2_, respectively, was associated with 2.20% (95%CI 1.0106–1.0335, *P* < 0.001), 0.24% (95% CI 1.0024–1.0166, *P* < 0.001), and 2.07% (95% CI 1.0106–1.0335, *P* < 0.001) increases in the risk of HF-related hospitalization or mortality. PM_2.5_ (1.29%, 95%CI 1.0093–1.0165, *P* < 0.001) and PM_10_ (1.30%, 95%CI 1.0102–1.0157, *P* < 0.001) were found to be positively associated with HF hospitalization or mortality (Supplementary Figures S1–S6). In addition, we conducted a subgroup analysis based on study design and age (Figure 3). There was no change in effect direction across all pollutants in these analyses (Supplementary Table S4).

### Sensitivity analysis and publication bias

Sensitivity analysis showed that the relationship between PM_2.5_ exposure and HF hospitalization or mortality was influenced by Amsalu et al. (16) (Supplementary Figure S13). The pooled standardized RR was changed to 1.0142 (95% CI: 1.0102–1.0182) after removing Amsalu's study. The association between PM_10_ exposure and HF hospitalization or mortality was affected by Morris et al. (19) (Supplementary Figure S14). After excluding, the pooled RR was changed to 1.0186 (1.0133–1.024). The sensitivity bias was also found in CO [Bell et al.'s (31), Supplementary Figure S16] and NO_2_ [Ye et al.'s (23), Supplementary Figure S15]. We recalculated the pooled RR and 95% CI after removing those studies (Supplementary Table S3). The sensitivity analysis of SO_2_ and O_3_ did not change by excluding each study, suggesting that the results were stable (Supplementary Figures S17, S18).

There was publication bias on studies of PM_2.5_, CO, and NO_2_ (Supplementary Figures S7, S10, S11), since the *p*-value of Begg's test was < 0.05. Other pollutants have no substantial publication bias (Egger's test, *p* > 0.05, Supplementary Figures 8, 9, 12). After using trimming and filling methods to adjust asymmetry, the effect value was slightly lower than that before correction, but the direction of effect estimation does not change (Table 2).

**Table 2:** Heterogeneity, population-attributable risk, and assessment for publication bias stratified by gaseous and particulate air pollutants.

	**Gaseous pollutants**				**Particulate matter**	
	**Carbon monoxide** **(ppm)**	**Nitrogen dioxide** **(ppb)**	**Sulfur dioxide** **(ppb)**	**Ozone** **(ppb)**	**PM**_2.5_ **(**μ**g/m**^3^**)**	**PM**_10_ **(**μ**g/m**^3^**)**
Increment	1 ppm	10 ppb	10 ppb	10 ppb	10 μg/m^3^	10 μg/m^3^
Median pollutant concentration (IQR)	1.14 (0.89–1.23)	16.45 (14.09–24.07)	2.24 (1.12–5.44)	36.28 (30.50–39.66)	47.15 (12.99–60.58)	64.86 (39.49–110.83)
Range (min–max)	0.20–1.49	12.55–41.37	0.98–11.79	20.2–43.60	2.9–102.1	36.47–131.50
Number of studies	20	18	19	19	28	27
Heterogeneity, *I*^2^	0.89	0.92	0.83	0.87	0.88	0.91
Population-attributable risk (PAR), % (95% CI)	3.34 (2.28–4.40)	2.63 (1.36–2.70)	2.15 (1.05–3.24)	0.94 (0.24–1.63)	1.27 (0.92–1.62)	1.28 (1.01–1.55)
**Publication bias**						
Egger regression test, *p*-value	0.001 (< 0.05)	0.002 (< 0.05)	0.132	0.143	0.000 (< 0.05)	0.078
Non-adjusted RR (95% CI)^§^	1.035 (1.023–1.046)	1.021 (1.014–1.028)	1.022 (1.011–1.034)	1.010 (1.002 −1.017)	1.013 (1.009–1.017)	1.013 (1.010–1.016)
Adjusted RR (95% CI)^¶^	1.018 (1.006–1.030)	1.008 (1.000–1.015)	/	/	1.008 (1.004–1.012)	/
Number of studies adjusted	8	8	/	/	8	/

ppb, parts per billion; PM, particulate matter; PAR, population-attributable risk; IQR, interquartile range. Median pollutant concentration (IQR) derived from the average daily pollutant concentrations reported per study. Range of the average pollutant concentrations across the studies from minimum to maximum. PAR reported per ten-unit increment in air pollutant concentration, except for CO where per one-unit increment. Calculated as PAR = X (RR – 1)/[X (RR – 1) + 1], where X indicates prevalence exposure (assumed to be 100% here).

§ Risk estimates derived from the pooled analysis of studies.

¶ Risk estimates after adjustment for publication bias using the trim and fill method.

## Discussion

This updated systematic review and meta-analysis enrolled 51 human epidemiological studies conducted in 11 countries. We found that the increase in four gas pollutants (CO, NO_2_, SO_2_, and O_3_) and two particulate matters (PM_2.5_ and PM_10_) was positively correlated with the HF hospitalization or mortality rate, regardless of the overall effect or lag effect. These results suggest that air pollution exposure was the risk factor for hospitalization or death in patients with HF. Among all the included pollutants, CO exposure had the greatest impact on the risk of hospitalization or death in patients with HF, while O_3_ exposure seemed to be the weakest one.

In 1995, Schwartz et al. (17) conducted the first human epidemiological study on air pollution exposure and the HF hospitalization rate in the United States and found that PM_10_ and CO, but not SO_2_ and O_3_, were positively correlated with HF. Since then, many studies explored the relationship between air pollution and HF. These studies showed inconsistent results. In 2013, a meta-analysis by Shah (13) showed that air pollution had a close temporal association with HF hospitalization and mortality. However, only one of the 35 studies was conducted in developing countries, making it difficult to assess the impact in developing countries. In Shah's study, exposures to PM_2.5_, PM_10_, SO_2_, NO_2_, and CO were positively correlated with HF hospitalization and mortality. However, exposure to ozone did not present this impact. Moreover, there were few studies on air pollution and HF in developing countries at that time. Only five studies in Shah's meta-analysis were from developing countries. In our meta-analysis, we updated the literature to 30 April 2022 and added 22 studies, including 14 studies from developing countries. We enrolled 7,555,442 cases, which was much more than the 3,374,700 participants in Shah's study (13). These made our evidentiary weight stronger.

Our results showed a high degree of heterogeneity, which might be due to the different pollutant detection methods and ethnicity and populations in different studies. Therefore, we aggregated estimates of effects based on standardized increments, which could help reduce heterogeneity. In a sensitivity analysis, we found that the direction remained unchanged and the correlation was positive after removing some studies.

Studies showed that PM_2.5_ is a critical factor for overall HF progression by regulating lung oxidative stress, inflammation, and RV remodeling (67). PM_2.5_ exposure triggered oxidative stress in the heart and systemic inflammation (68) and inhibited vascular endothelial repair capacity (69). It was reported that air pollution aggravated aortic endothelial dysfunction in HF rats (69). In addition, CO aggressively binds to hemoglobin with an affinity 200 times greater than that of oxygen, resulting in a reduced fraction of oxygenated hemoglobin in the bloodstream (70). This weakens the blood's capacity to deliver oxygen to the tissues (71). Thus, chronic exposure to high levels of CO will induce tissue hypoxia. Some studies indicated that exposure to SO_2_ was sufficient to disrupt excitatory synaptic inputs to cardiac vagal neurons, the reflexive control part of heart rate, and induce tachycardia (72). Taken together, many studies reported the relationship between air pollution and HF. However, the molecular mechanism is still ambiguous.

There were several limitations to our study. First, part of the data came from routine administrative sources, which might introduce bias due to coding errors and misclassification. Second, there were three kinds of pollutants (PM_2.5_, SO_2_, and NO_2_) that had publication bias despite the direction of the adjusted overall effect remaining unchanged. Third, we found significant heterogeneity across all pollutants, which indicated the differences in population demographics, sample size, and patient characteristics.

Heart failure is a common and fatal disease. We found that exposure to all pollutants, including ozone, was positively associated with HF hospitalization and mortality. However, due to the limited number of studies on short-term effects, caution should still be taken in interpreting our results.

## Data availability statement

The original contributions presented in the study are included in the article/Supplementary material, further inquiries can be directed to the corresponding authors.

## Author contributions

X-hC, PY, and Y-hP designed the study, coordinated the study, and directed its implementation. Y-sY and Y-yG collected data and conducted the follow-up work. Y-sY and J-fZ wrote the manuscript. All authors have read and approved the final manuscript.
