# Supplementary material for: Association between short-term exposure to ambient air pollution and heart failure: An updated systematic review and meta-analysis of more than 7 million participants
Source: Front Public Health. 2023 Jan 23;10:948765. doi: 10.3389/fpubh.2022.948765 (PMC9900180; doi:10.3389/fpubh.2022.948765)
Supplement: Supplementary file 1 [file Data_Sheet_1.docx]

Supplementary materials

**Table S1.** PRISMA 2009 Checklist

| **Section/topic** | **#** | **Checklist item** | **Reported on page #** | |
| --- | --- | --- | --- | --- |
| **TITLE** | | |  | |
| Title | 1 | Identify the report as a systematic review, meta-analysis, or both. | Page 1 | |
| **ABSTRACT** | | |  | |
| Structured summary | 2 | Provide a structured summary including, as applicable: background; objectives; data sources; study eligibility criteria, participants, and interventions; study appraisal and synthesis methods; results; limitations; conclusions and implications of key findings; systematic review registration number. | Page1,2 | |
| **INTRODUCTION** | | |  | |
| Rationale | 3 | Describe the rationale for the review in the context of what is already known. | Page 2 | |
| Objectives | 4 | Provide an explicit statement of questions being addressed with reference to participants, interventions, comparisons, outcomes, and study design (PICOS). | Page 3 | |
| **METHODS** | | |  | |
| Protocol and registration | 5 | Indicate if a review protocol exists, if and where it can be accessed (e.g., Web address), and, if available, provide registration information including registration number. | Page 3 | |
| Eligibility criteria | 6 | Specify study characteristics (e.g., PICOS, length of follow-up) and report characteristics (e.g., years considered, language, publication status) used as criteria for eligibility, giving rationale. | Page 3 | |
| Information sources | 7 | Describe all information sources (e.g., databases with dates of coverage, contact with study authors to identify additional studies) in the search and date last searched. | Page 3 | |
| Search | 8 | Present full electronic search strategy for at least one database, including any limits used, such that it could be repeated. | Page3 | |
| Study selection | 9 | State the process for selecting studies (i.e., screening, eligibility, included in systematic review, and, if applicable, included in the meta-analysis). | Page3 | |
| Data collection process | 10 | Describe method of data extraction from reports (e.g., piloted forms, independently, in duplicate) and any processes for obtaining and confirming data from investigators. | Page 3,4 | |
| Data items | 11 | List and define all variables for which data were sought (e.g., PICOS, funding sources) and any assumptions and simplifications made. | NA | |
| Risk of bias in individual studies | 12 | Describe methods used for assessing risk of bias of individual studies (including specification of whether this was done at the study or outcome level), and how this information is to be used in any data synthesis. | Page4,5 | |
| Summary measures | 13 | State the principal summary measures (e.g., risk ratio, difference in means). | Page 4 | |
| Synthesis of results | 14 | Describe the methods of handling data and combining results of studies, if done, including measures of consistency (e.g., I2) for each meta-analysis. | Page 5 | |
| **Section/topic** | **#** | **Checklist item** | **Reported on page #** |  |
| Risk of bias across studies | 15 | Specify any assessment of risk of bias that may affect the cumulative evidence (e.g., publication bias, selective reporting within studies). | Page5 |  |
| Additional analyses | 16 | Describe methods of additional analyses (e.g., sensitivity or subgroup analyses, meta-regression), if done, indicating which were pre-specified. | Page 5 |  |
| **RESULTS** | | |  |  |
| Study selection | 17 | Give numbers of studies screened, assessed for eligibility, and included in the review, with reasons for exclusions at each stage, ideally with a flow diagram. | Page 5 |  |
| Study characteristics | 18 | For each study, present characteristics for which data were extracted (e.g., study size, PICOS, follow-up period) and provide the citations. | Page 5 |  |
| Risk of bias within studies | 19 | Present data on risk of bias of each study and, if available, any outcome level assessment (see item 12). | Page 6 |  |
| Results of individual studies | 20 | For all outcomes considered (benefits or harms), present, for each study: (a) simple summary data for each intervention group (b) effect estimates and confidence intervals, ideally with a forest plot. | Page 6 |  |
| Synthesis of results | 21 | Present results of each meta-analysis done, including confidence intervals and measures of consistency. | Page 6 |  |
| Risk of bias across studies | 22 | Present results of any assessment of risk of bias across studies (see Item 15). | Page 6 |  |
| Additional analysis | 23 | Give results of additional analyses, if done (e.g., sensitivity or subgroup analyses, meta-regression [see Item 16]). | Page 6 |  |
| **DISCUSSION** | | |  |  |
| Summary of evidence | 24 | Summarize the main findings including the strength of evidence for each main outcome; consider their relevance to key groups (e.g., healthcare providers, users, and policy makers). | Page 6,7 |  |
| Limitations | 25 | Discuss limitations at study and outcome level (e.g., risk of bias), and at review-level (e.g., incomplete retrieval of identified research, reporting bias). | Page 8 |  |
| Conclusions | 26 | Provide a general interpretation of the results in the context of other evidence, and implications for future research. | Page 6-8 |  |
| **FUNDING** | | |  |  |
| Funding | 27 | Describe sources of funding for the systematic review and other support (e.g., supply of data); role of funders for the systematic review. | Page 8 |  |

*From:*  Moher D, Liberati A, Tetzlaff J, Altman DG, The PRISMA Group (2009). Preferred Reporting Items for Systematic Reviews and Meta-Analyses: The PRISMA Statement. PLoS Med 6(7): e1000097. doi:10.1371/journal.pmed1000097

For more information, visit: **www.prisma-statement.org**.

**Table S2.** Quality assessment of included studies

| Reference | Quality assessment (NOS) | | |  |
| --- | --- | --- | --- | --- |
|  | Selection | Comparability | Outcome | Total  score |
| Schwartz et al.1995 | 3 | 2 | 3 | 8 |
| Poloniecki et al.1997 | 4 | 1 | 3 | 8 |
| Morris et al.1998 | 3 | 1 | 3 | 7 |
| Wong TW et al.1999 | 4 | 2 | 2 | 8 |
| Wong CM et al.1999 | 4 | 2 | 3 | 9 |
| Hoek et al.2001 | 3 | 2 | 2 | 7 |
| Kwon et al.2001 | 4 | 2 | 3 | 9 |
| Ye et al.2001 | 3 | 2 | 3 | 8 |
| McGowan et al.2002 | 4 | 2 | 3 | 9 |
| Goldberg et al.2003 | 4 | 2 | 3 | 9 |
| Koken et al.2003 | 4 | 2 | 3 | 9 |
| Bateson et al.2004 | 3 | 2 | 3 | 8 |
| Metzger et al.2004 | 4 | 1 | 3 | 8 |
| Wellenius et al.2005 | 4 | 1 | 3 | 8 |
| Martins et al.2006 | 4 | 1 | 2 | 7 |
| Dominici et al.2006 | 4 | 1 | 3 | 8 |
| Wellenius et al.2006 | 4 | 2 | 3 | 9 |
| Barnett et al.2006 | 4 | 1 | 3 | 8 |
| Lee et al.2007 | 3 | 2 | 3 | 8 |
| Peel et al.2007 | 4 | 2 | 3 | 9 |
| Forastiere et al.2008 | 3 | 1 | 3 | 7 |
| Yang et al.2008 | 4 | 1 | 3 | 8 |
| Bell et al.2009 | 4 | 2 | 3 | 9 |
| Haley et al.2009 | 3 | 2 | 3 | 8 |
| Stieb et al.2009 | 4 | 2 | 3 | 9 |
| Ueda et al.2009 | 3 | 2 | 3 | 8 |
| Zanobetti et al.2009 | 4 | 2 | 3 | 9 |
| Colais et al.2009 | 4 | 2 | 3 | 9 |
| Belleudi et al.2010 | 4 | 2 | 3 | 9 |
| Hsieh et al .2013 | 3 | 2 | 3 | 8 |
| Yang et al.2014 | 4 | 1 | 2 | 7 |
| Milojevic et al .2014 | 4 | 1 | 2 | 7 |
| Chen et al.2015 | 3 | 2 | 3 | 8 |
| Weber et al.2016 | 4 | 2 | 2 | 8 |
| Vaduganathan et al.2016 | 4 | 2 | 3 | 9 |
| Dabass et al.2016 | 3 | 2 | 3 | 8 |
| Xu et al.2017 | 4 | 2 | 3 | 9 |
| Liu et al.2017 | 3 | 2 | 3 | 8 |
| Hsu et al .2017 | 4 | 2 | 3 | 9 |
| Li et al.2018 | 4 | 2 | 3 | 9 |
| Huynh et al.2018 | 4 | 2 | 2 | 8 |
| Li et al.2018 | 3 | 1 | 3 | 7 |
| Zhang et al.2018 | 4 | 1 | 3 | 8 |
| Pothirat et al .2019 | 4 | 1 | 3 | 8 |
| Amsalu et al.2019 | 4 | 1 | 2 | 7 |
| Tian et al.2019 | 4 | 2 | 3 | 9 |
| Wu et al.2019 | 4 | 1 | 3 | 7 |
| Feng et al.2019 | 4 | 2 | 3 | 9 |
| Qiu et al.2020 | 3 | 2 | 3 | 8 |
| Gu et al.2020 | 4 | 1 | 3 | 8 |
| Pamplona et al.2020 | 3 | 1 | 3 | 7 |

| **Table S3.** Sensitivity analysis of the association between exposure to air pollution and HF removing influenced studies | | | | | | |
| --- | --- | --- | --- | --- | --- | --- |
| Air pollutants | Before removing influenced studies | |  | After removing influenced studies | |  |
|  | NO. | RR |  | NO. | RR |  |
| CO | 20 | 1.0346(1.0233-1.046) | 19 | | 1.038(1.0254-1.0507) |  |
| NO_2_ | 19 | 1.027(1.0138-1.0277) | 18 | | 1.0236(1.0159-1.0315) |  |
| PM_10_ | 27 | 1.0130(1.0102-1.0157) | 26 | | 1.0179(1.0129-1.0229) |  |
| PM_2.5_ | 28 | 1.0129(1.0093-1.0165) | 27 | | 1.0142(1.0102-1.0182) |  |

**Table S4.** Subgroup analysis of the association between exposure to air pollution and HF

| Air pollutants - HF | Subgroup | | NO. | RR | I2/P value |
| --- | --- | --- | --- | --- | --- |
| CO （1ppm） | Age | all | 11 | 1.045 (1.021 - 1.070) | 84.3%/<0.001 |
|  |  | ≥60 | 9 | 1.034 (1.022 - 1.046) | 84.4%/<0.001 |
|  | study designs | case-crossover | 6 | 1.075 (1.030 - 1.122) | 91.7%/<0.001 |
|  |  | time series | 14 | 1.025 (1.015 - 1.036) | 85.7%/<0.001 |
|  | outcomes | hospitalization | 16 | 1.034 (1.021 - 1.045) | 90.2%/<0.001 |
|  |  | mortality | 4 | 1.039 (1.029 – 1.049) | 0/0.454 |
| SO2  （10ppb） | Age | all | 9 | 1.032(0.997 – 1.068) | 78.5%/<0.001 |
|  |  | ≥60 | 9 | 1.020 (1.008 - 1.032) | 87.4%/<0.001 |
|  | study designs | case-crossover | 4 | 1.005 (0.981 – 1.031) | 64.9%/0.036 |
|  |  | time series | 14 | 1.027 (1.014 – 1.041) | 86%/<0.001 |
|  | outcomes | hospitalization | 4 | 1.018 (1.005 – 1.031) | 82.9%/<0.001 |
|  |  | mortality | 14 | 1.053 (1.008 – 1.100) | 80.4%/0.006 |
| NO2 （10ppb） | Age | all | 11 | 1.045 (1.021 - 1.070) | 89%/<0.001 |
|  |  | ≥60 | 8 | 1.011 (1.004 - 1.018) | 88.2%/<0.001 |
|  | study designs | case-crossover | 5 | 1.062 (1.012 – 1.115) | 94.3%/<0.001 |
|  |  | time series | 14 | 1.016 (1.009 – 1.022) | 90.9%/<0.001 |
|  | outcomes | hospitalization | 15 | 1.021 (1.014 – 1.029) | 93.4%/<0.001 |
|  |  | mortality | 4 | 1.020 (1.001 – 1.039) | 66%/0.032 |
| O3 （10ppb） | Age | all | 9 | 1.038 (1.010 - 1.067) | 91.9%/<0.001 |
|  |  | ≥60 | 10 | 1.002 (0.997 - 1.067) | 78.3%/<0.001 |
|  | study designs | case-crossover | 7 | 0.999 (1.023 - 1.048) | 93.8%/<0.001 |
|  |  | time series | 12 | 1.005 (0.999 - 1.011) | 70.8%/<0.001 |
|  | outcomes | hospitalization | 15 | 1.010(1.001 – 1.020) | 89.4%/<0.001 |
|  |  | mortality | 4 | 1.009 (0.998 – 1.020) | 38.7%/0.18 |
| PM2.5 （10μg/m3） | Age | all | 20 | 1.011 (1.008 - 1.015) | 89.5%/<0.001 |
|  |  | ≥60 | 7 | 1.016 (1.010 - 1.022) | 54.8%/0.039 |
|  | study designs | case-crossover | 15 | 1.020 (1.013 - 1.026) | 90.3%/<0.001 |
|  |  | time series | 12 | 1.007 (1.004 - 1.011) | 82.6%/<0.001 |
|  | outcomes | hospitalization | 22 | 1.013 (1.010 - 1.017) | 90.8%/<0.001 |
|  |  | mortality | 5 | 1.008 (0.992 – 1.025) | 0/0.571 |
| PM10 （10μg/m3） | Age | all | 17 | 1.020 (1.011 - 1.028) | 89.7%/<0.001 |
|  |  | ≥60 | 11 | 1.011 (1.007 - 1.015) | 93.4%/<0.001 |
|  | study designs | case-crossover | 13 | 1.021 (1.012 – 1.030) | 93.7%/<0.001 |
|  |  | time series | 15 | 1.010 (1.006 – 1.013) | 81.7%/<0.001 |
|  | outcomes | hospitalization | 22 | 1.016 (1.012 – 1.019) | 92.9%/<0.001 |
|  |  | mortality | 6 | 1.006 (1.001 – 1.011) | 43.9%/0.112 |


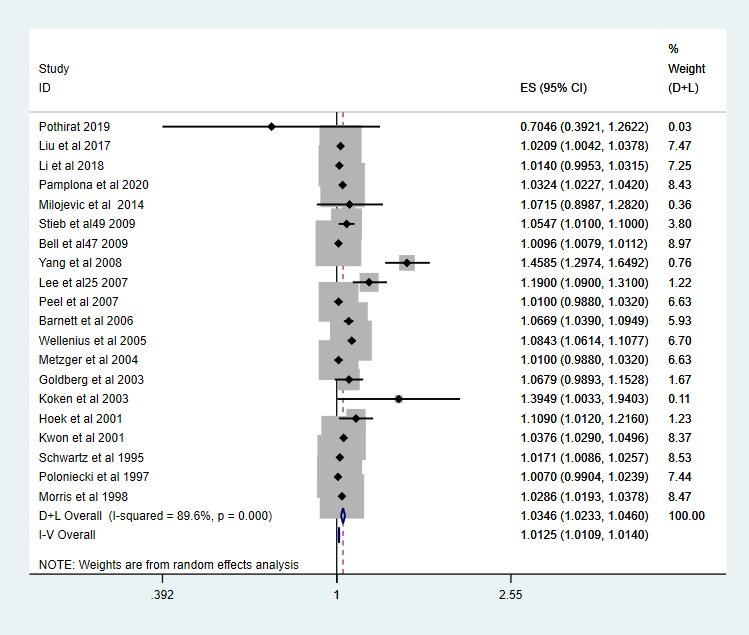


**Figure S1**. Meta-analysis of HF and exposure to CO for increments of 1ppm.


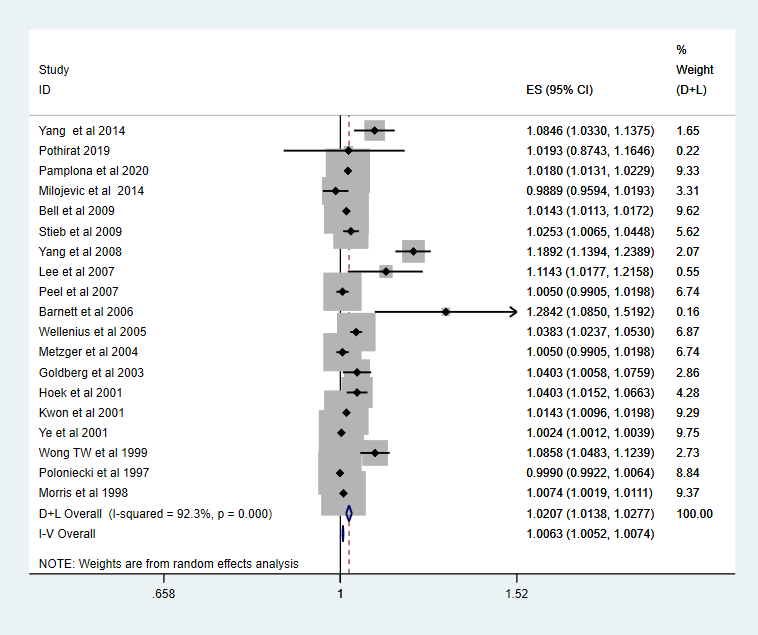


**Figure S2**. Meta-analysis of HF and exposure to NO_2_ for increments of 10 ppb.


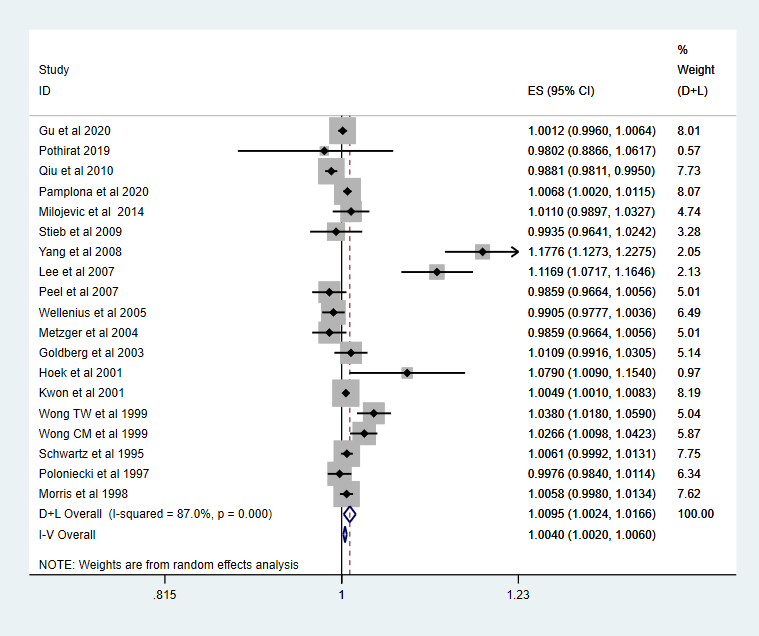


**Figure S3**. Meta-analysis of HF and exposure to O_3_ for increments of 10ppb.


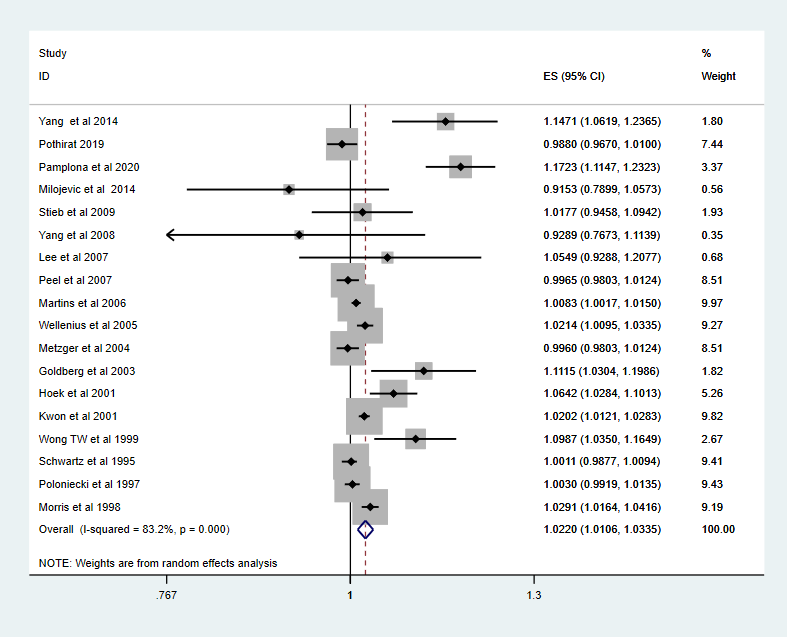


**Figure S4**. Meta-analysis of HF and exposure to SO_2_ for increments of 10ppb.


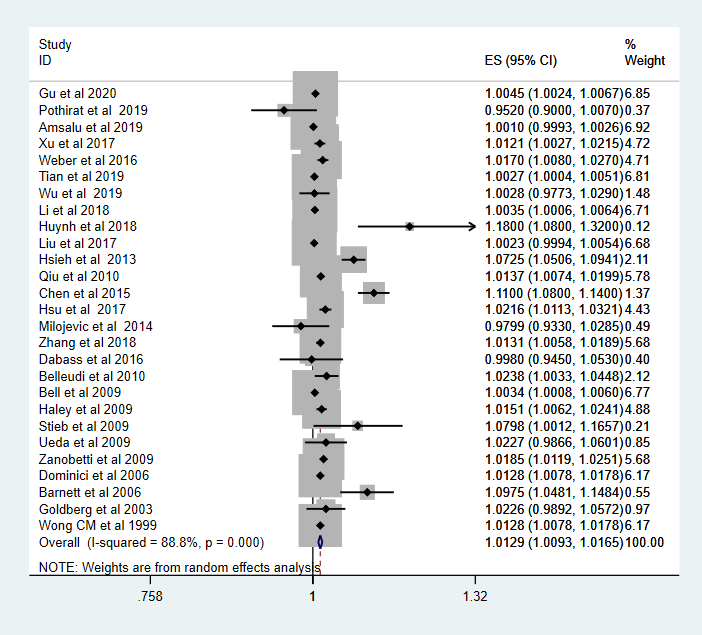


**Figure S5**. Meta-analysis of HF and exposure to PM_2.5_ for increments of 10 μg/m^3^.


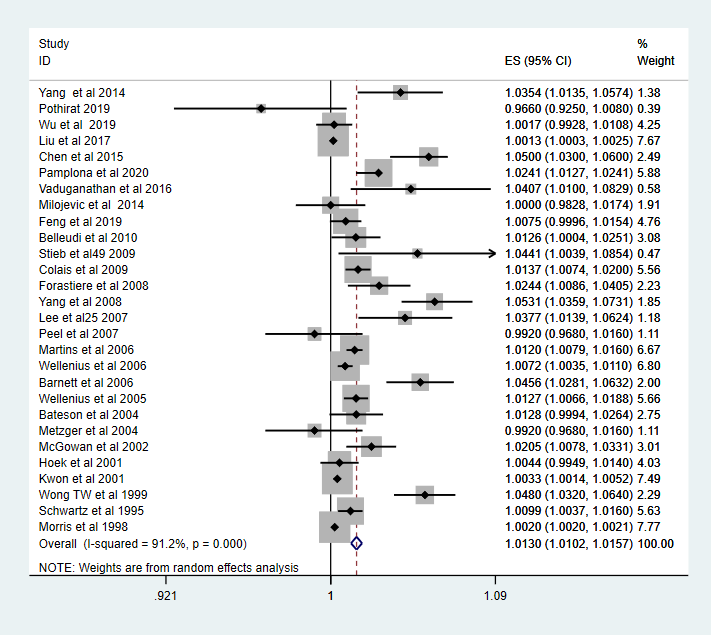


**Figure S6**. Meta-analysis of HF and exposure to PM_10_ for increments of 10 μg/m^3^.


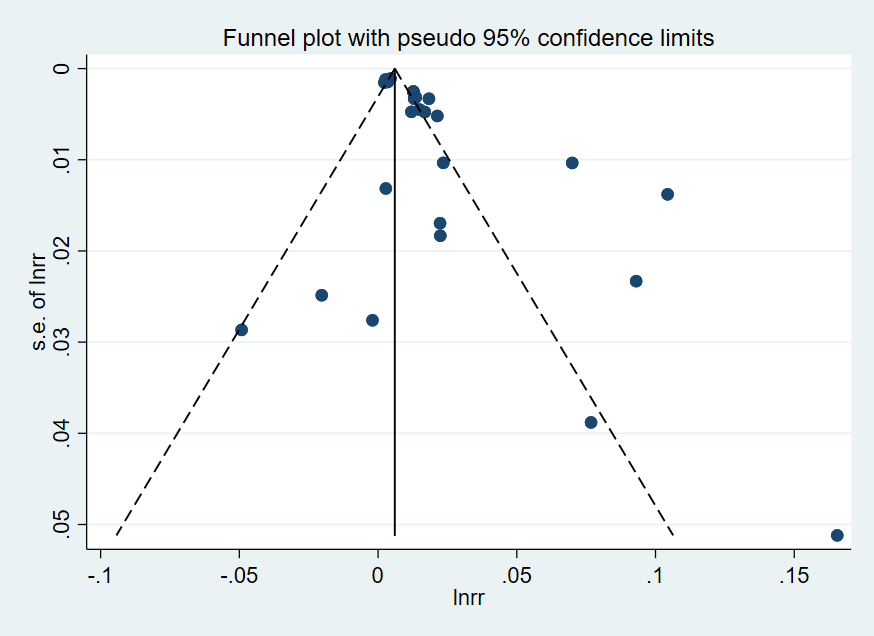


**Figure S7.** Funnel plot analysis on the detection of publication in the meta-analysis of the association between PM_2.5_ and HF


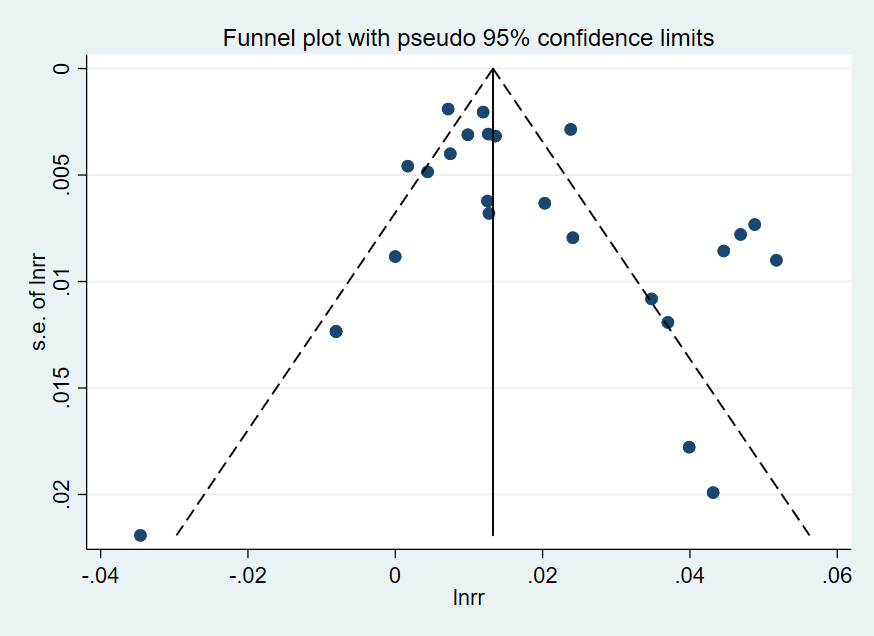


**Figure S8.** Funnel plot analysis on the detection of publication in the meta-analysis of the association between PM_10_ and HF

**
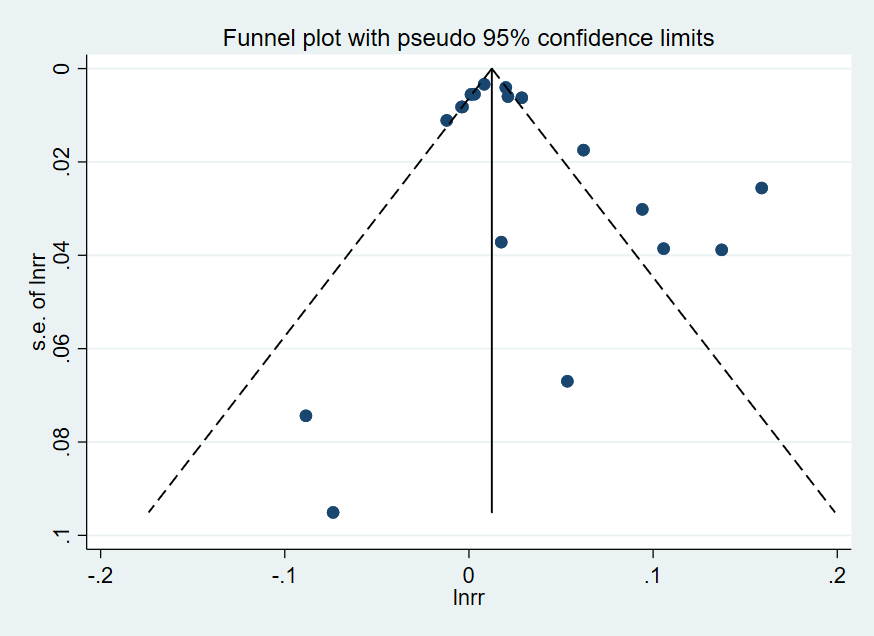
**

**Figure S9.** Funnel plot analysis on the detection of publication in the meta-analysis of the association between SO_2_ and HF

**
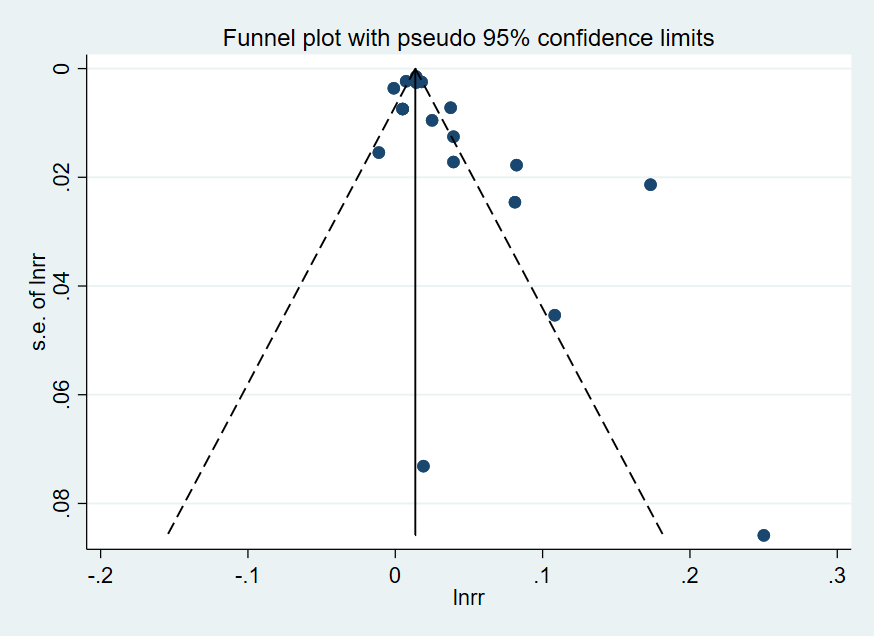
**

**Figure S10.** Funnel plot analysis on the detection of publication in the meta-analysis of the association between NO_2_ and HF

**
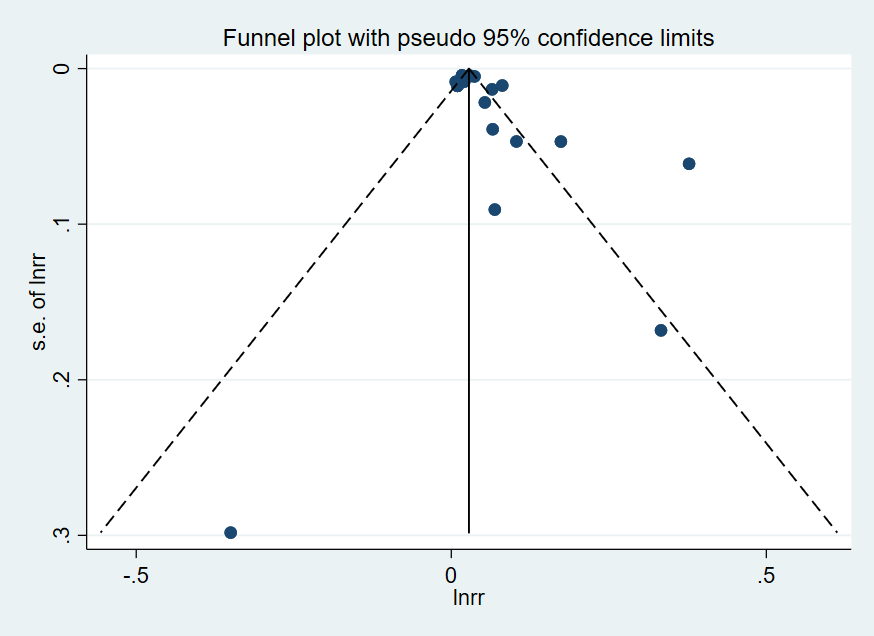
**

**Figure S11.** Funnel plot analysis on the detection of publication in the meta-analysis of the association between CO and HF

**
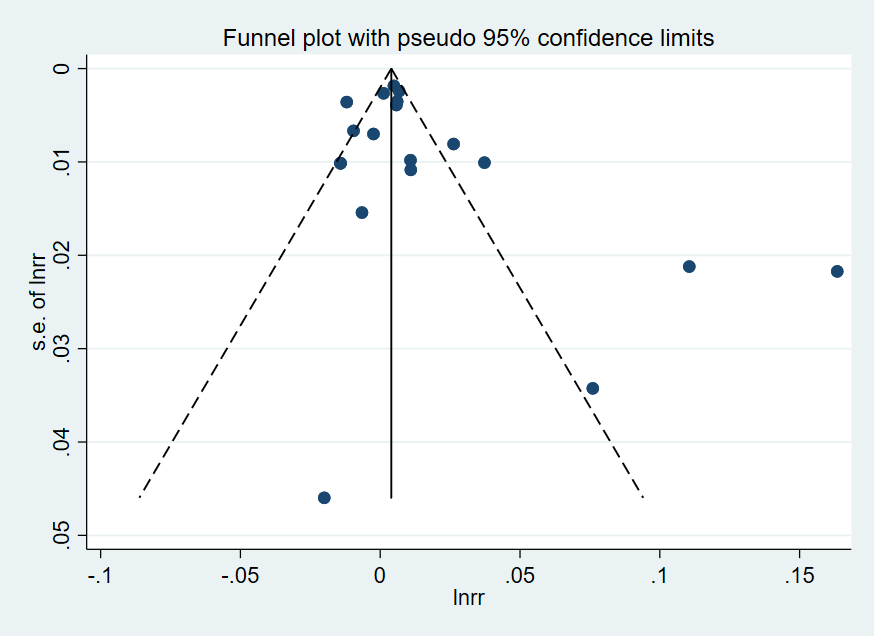
**

**Figure S12.** Funnel plot analysis on the detection of publication in the meta-analysis of the association between O_3_ and HF

**Figure S13.**Sensitivity analysis of the association between PM_2.5_ and HF by excluding each study

**Figure S14.**Sensitivity analysis of the association between PM_10_ and HF by excluding each study

**Figure S15.**Sensitivity analysis of the association between NO_2_ and HF by excluding each study

**Figure S16.**Sensitivity analysis of the association between CO and HF by excluding each study

**Figure S17.**Sensitivity analysis of the association between O_3_ and HF by excluding each study

**Figure S18.**Sensitivity analysis of the association between SO_2_ and HF by excluding each study
